# The Risk of Oral Cancer and the High Consumption of Thermally Processed Meat Containing Mutagenic and Carcinogenic Compounds

**DOI:** 10.3390/nu16071084

**Published:** 2024-04-07

**Authors:** Sylwia Bulanda, Karolina Lau, Agnieszka Nowak, Dorota Łyko-Morawska, Anna Kotylak, Beata Janoszka

**Affiliations:** 1 Department of Chemistry, Faculty of Medical Sciences in Zabrze, Medical University of Silesia in Katowice, Jordana 19, 41-808 Zabrze, Poland; agnieszkanowak@sum.edu.pl; 2 Department of Environmental Medicine and Epidemiology in Zabrze, Medical University of Silesia in Katowice, Jordana 19, 41-808 Zabrze, Poland; karolina.lau@sum.edu.pl; 3 Department of Vascular Surgery, General Surgery, Angiology and Phlebology, Medical University of Silesia, Ziołowa 45/47, 40-635 Katowice, Poland; dorota.lyko@sum.edu.pl; 4 I Radiation and Clinical Oncology Department, Maria Skłodowska-Curie National Research Institute of Oncology, Gliwice Branch, 44-102 Gliwice, Poland; anna.kotylak@gliwice.nio.gov.pl

**Keywords:** oral cancer, thermally processed meat, red meat, FFQ, heterocyclic aromatic amines, polycyclic aromatic hydrocarbons

## Abstract

The International Agency for Research on Cancer has classified the consumption of heat-processed meat as a direct human carcinogen and the consumption of red meat as a probable carcinogen. Mutagenic and carcinogenic compounds present in meat dishes include, among others, polycyclic aromatic hydrocarbons (PAHs) and heterocyclic aromatic amines (HAAs). These compounds can cause the development of gastrointestinal cancer. Oral cancer is one of the world’s research priorities due to the ever-increasing incidence rate. However, the effect of diet on oral cancer is still a poorly recognized issue. The aim of this study was to assess the relationship between the risk of oral cancer and dietary ingredients with a particular emphasis on red meat and thermally processed meat. This study was conducted among patients with oral cancer in 2022 and 2023. The shortened standardized Food Frequency Questionnaire (FFQ) and a multivariate regression statistical analysis were used. The high consumption of red meat in general and thermally processed meat, especially smoked, fried, roasted and boiled, increases the risk of oral cavity cancer. Limiting the consumption of meat products and modifying the methods of preparing meat dishes may reduce exposure to carcinogenic compounds from the diet and thus reduce the risk of developing oral cancer.

## 1. Introduction

Epidemiological research indicates that 30–35% of cancers are diet-related [1]. Food contains many chemical compounds harmful to humans. They may come from the environment, the production process, or are formed during the storage and thermal processing of food [1]. They are often mutagenic and carcinogenic compounds, which are formed in large quantities in high-protein products. These include, among others, polycyclic aromatic hydrocarbons (PAHs) and heterocyclic aromatic amines (HAAs) [2].

In 2015, the International Agency for Research on Cancer (IARC) classified frequent consumption of red meat as “probably carcinogenic to humans” (carcinogenicity group 2A) and the consumption of processed meat, including smoked and heat-treated meat, as “directly carcinogenic” (carcinogenicity group 1) [3].

Dietary habits influence the development of various types of cancer [4]. Some dietary components may increase or decrease inflammation in the body. A pro-inflammatory diet causes persistent inflammation, promoting the development of cancer in some parts of the body [5]. The influence of diet on the development of oral cancer is an issue that has not been intensively studied so far, so this study attempts to assess whether frequent consumption of meat products may be a risk factor for oral cancer.

Oral cell carcinomas (OCCs) are lesions occurring on the mucous membrane of the lips, the anterior part (3/4) of the tongue, the cheek, the floor of the mouth, the gingiva, the hard palate, the alveolar process and the retromolar triangle [6]. They are part of head and neck cancers, which is a large group of cancers located in the area above the collarbones, excluding cancers of the central nervous system and the eyeball. About one-third of head and neck cancers are located on the tongue—usually in its front part and usually on the lateral and lower surfaces. The second most common is oral floor cancer, and the third most common is gingiva cancer [6]. OCCs are mainly (90%) a squamous cell carcinoma originating from the stratified squamous epithelium [4,7,8]. There is an increasing trend in the incidence of oral cancer worldwide [6]. The risk of developing the disease increases with age. Most cases occur in the fifth decade of life; however, 6% of patients are under 45 years of age [6,9].

The main confirmed risk factors for the development of oral cancer include smoking cigarettes and the use of other tobacco products, drinking alcohol and infection with the human papillomavirus (HPV). However, many patients develop oral cancer despite no exposure to the above risk factors. The disease is then determined by genetic susceptibility, environmental factors, UV radiation, ethnicity, oral hygiene, chronic mechanical irritation (e.g., poorly fitting prosthetic restorations), occupational risk and diet [10].

One way that diet may increase the risk of oral cancer is through the formation of inflammatory biomarkers such as CRP, IL-6 and homocysteine. The inflammatory process is responsible for the delivery of bioactive molecules to the tumor environment. Furthermore, inflammatory transcription factors can be activated by cytokines and other inflammatory biomarkers, influencing the cancer initiation and promotion [11].

The food groups that increase the risk of cancer are mainly processed red meat [10]. Processed meat has an important role in the development of cancer due to its high content of saturated fat and heme iron, as well as strong mutagens. The increased risk associated with the consumption of meat products may result from exposure to carcinogenic compounds, including heterocyclic aromatic amines and polycyclic aromatic hydrocarbon, generated during meat processing at high temperatures. The compounds found in thermally processed protein-rich products may disturb the synthesis of DNA, increase cell proliferation, influence hormone metabolism, increase the concentration of insulin growth factors (IGF-1), and contribute to the formation of free radicals, intensifying the processes of carcinogenesis [4,5].

Polycyclic aromatic hydrocarbons are a group of more than 200 organic compounds that are formed during the incomplete combustion of organic matter [12]. Due to their lipophilic properties, they can accumulate in the food chain [13]. An important source of PAHs in the diet are protein products (fish and meat), which, in order to preserve and improve their taste, are subjected to smoking or other high-temperature processes, e.g., grilling, baking, and frying [14,15,16].

In accordance with the applicable Commission Regulation (EU) No. 835/2011, “smoked meat and meat products” as well as commercially available “meat and meat products heat treated by roasting on a grill” should not contain more of the carcinogenic hydrocarbon benzo(a) pyrene (BaP) than 5 µg/kg, and the total concentration of four compounds (PAH4)—BaP, benz(a)anthracene [BaA], benzo(b)fluoranthene [BbFl] and chrysene [Chr]—should be less than 30 µg/kg. In 2014, these levels were reduced to 2 µg/kg and 12 µg/kg, respectively. However, in some EU countries this lower level of PAH content was not achievable in traditionally smoked meat products, so higher concentration standards for BaP and PAH4 were reintroduced. The concentration of PAHs in meat products depends largely on the method of preparation of the dishes. The heat treatment methods involving grilling, mainly on charcoal, usually result in products with a higher PAH content than those obtained by frying, roasting, or cooking the meat. The content of the individual PAH compounds in the meat subjected to heat treatment is usually from 0 to several µg/kg, although, according to some research results, the concentrations of PAH4 and BaP may be much higher and even reach values close to 200 µg/kg and 65 µg/kg (in barbecued meat) [4,17]. One of the reasons for the presence of large amounts of PAHs in grilled and smoked meat is the deposition on its surface of the products of incomplete combustion of rendered fat and other meat ingredients that have entered the furnace [17]. This situation does not occur when cooking and baking (stewing) meat in a pan or using a baking sleeve [4]. During smoking, a portion of meat may additionally accumulate PAHs formed as a result of incomplete combustion of the wood used to produce smoke.

The second group of carcinogenic compounds that are formed in high-protein products are heterocyclic aromatic amines, which are organic nitrogen compounds composed of two or three fused rings, one of which is aromatic and the rest are heterocyclic. Except for three compounds from this group, all of them have one exocyclic amino group (–NH_2_) [18,19,20]. They are formed in the Maillard reaction from naturally occurring α-amino acids, sugars (e.g., glucose and fructose) and creatine [18,21].

The maximum allowable levels of these compounds in food have not yet been established. The type and concentration of heterocyclic aromatic amines formed in food products depend on many factors, primarily the temperature, heating time and method of heat treatment [20,22,23,24]. Moreover, they depend on the type of meat and its content of amino acids [25,26], sugars and creatine [18]; on the type of fats used for the frying [26]; and the additives used to prepare the dish [18,20,21,27].

Heterocyclic aromatic amines are formed after just a few minutes of the heat treatment of meat and are present even in dishes with a slight degree of roasting [18,21]. The concentrations of individual HAAs in meat dishes range from 0 to several dozen µg in 1 kg of product. Grilled meat, as well as meat fried for a long time at a high temperature, contains more HAAs than dishes cooked or prepared by baking, e.g., in an electric oven [18,20,21,22,23,24,26,27]. So far, over 30 compounds from the group of heterocyclic amines have been detected. The total content of HAAs in meat products may be higher than the content of PAHs [4]. 

It is worth adding that compounds from the HAA group, although not classified by the International Agency for Research on Cancer as directly carcinogenic to humans, like BaP, are characterized by much stronger mutagenic activity [28]. 

Polycyclic aromatic hydrocarbons and heterocyclic aromatic amines are compounds whose mutagenic and genotoxic properties are revealed as a result of metabolic activation under the influence of cytochrome P450 (CYP450) enzymes. Carcinogenic compounds from PAHs may be formed as a result of reactions taking place via three main pathways. The first pathway involving cytochrome peroxidase leads to the formation of cation radicals (a radical cation of PAHs). The second one, which takes place with the participation of cytochrome CYP1A1/1B1 and epoxide hydrolase, leads to the formation of diolepoxides of PAHs. The third pathway is catalyzed by aldo-keto reductase and leads to the formation of o-quinones of PAHs. The resulting products of the metabolic activation of PAHs can form adducts with DNA molecules. Their presence in the body may result in errors in the DNA replication processes and disruptions in the methylation process or promoter binding. DNA mutations or abnormal gene expression may ultimately lead to cancer [29]. PAH metabolites also form adducts with proteins, which may result in modification of their activity. Moreover, the reactive oxygen species generated by PAH metabolites may initiate carcinogenesis by modifying DNA, proteins and lipids [29]. 

Heterocyclic aromatic amines, due to the presence of an exocyclic amine group in their molecules, are among the most biologically active compounds identified so far in food. HAAs, like PAHs, are metabolically activated with the catalytic participation of cytochrome P450 enzymes. Initially, N-hydroxylation of the exocyclic amino group to hydroxyamine (–NHOH) occurs. Subsequently, under the influence of enzymes from the group of sulfotransferases and N-acetyltransferases, very active metabolites (sulfates and esters) are formed. They can combine with DNA molecules and protein molecules. Moreover, a metabolite containing an acetyl or sulfate group may produce a reactive diazonium ion, capable of forming an adduct with DNA [15,22]. The resulting stable adducts can cause errors in DNA replication, which in turn can lead to mutations and then to the development of cancer.

A review of the literature on epidemiological studies and the meta-analyses on exposure to PAHs and HAAs shows that frequent consumption of heat-treated meat products may lead to the development of cancer in various areas of the human body [30].

The aim of this study was to statistically evaluate the association between oral cancer risk and dietary components. The authors wanted to check whether high consumption of meat (especially red meat) and thermally processed meat are risk factors for oral cancer. In this research, particular attention was paid to the risk of carcinogenicity associated with the consumption of roasted, fried, grilled, boiled, and smoked meat. Additionally, a goal was to assess whether products with antioxidant properties (fruits and vegetables) can help reduce the risk of oral cancer.

## 2. Materials and Methods

### 2.1. Characteristics of the Study and Control Groups

This research was an observational study. It was conducted among oncology patients in the form of a direct interview.

There were 165 people that were included in this study. The study group included 85 people diagnosed with oral cancer. These were patients diagnosed with C00-C08 and C14 according to the ICD-10 classification. These were people hospitalized at the National Institute of Oncology M. Skłodowska-Curie in Gliwice, Poland, from January 2022 to March 2023. The study included patients of both sexes, over 18 years of age, with a primary diagnosis of oral cancer who consented to participate in the study. Due to communication difficulties, people who had undergone extensive surgical procedures, people struggling with their mental health, and patients with chronic diseases (e.g., diabetes and kidney failure) were excluded from this study. These were people aged 26–85 (average age 67.5).

The control group consisted of adults of both sexes who agreed to participate in this study. According to the information provided in the survey, these people were not diagnosed with cancer or chronic diseases. These were people aged 23–84 (average age 53).

During this study, the patient data and disease histories included in the clinical cards provided by the National Institute of Oncology in Gliwice were analyzed.

The groups were selected according to gender, demographic distribution, and age. All study participants were living in one area in Poland (Silesia).

The study protocol was approved by the ethical committees of the Medical University of Silesia in Katowice, Poland, nr PNC/CBN/0022/KB/266/21.

### 2.2. Source of Diet Information: Food Frequency Questionnaire (FFQ)

The information on the frequency of the consumption of specific food products was obtained during this study using a standardized Food Frequency Questionnaire (FFQ). Additionally, sociodemographic factors were analyzed, such as age, gender, profession, place of residence, and the impact of smoking and drinking alcohol.

The original version of the FFQ contains questions about 165 types of food, which have been assigned to several food groups and makes it possible to qualitatively and quantitatively assess the respondents’ diet. This study used a shortened version of the questionnaire covering 33 products divided into six groups (Table 1), which assesses the diet in a qualitative and semi-quantitative way. The FFQ-6 was validated and translated into Polish by Wądołowska et al. [31]. The respondents were asked to choose one of six answers regarding the frequency of consumption of specific products in the 12 months before the first symptoms of cancer appeared. The frequency of consumption of specific products had to be classified into specific ranges: 1—never, 2—once a month, 3—several times a month, 4—several times a week, 5—every day, and 6—several times a day.

### 2.3. Method Limitations

The above method has some limitations. In the survey, respondents answer only the questions provided in the questionnaire. The short questions limit the ability to delve deeper into the research problem. During surveys, we obtain specific data that can only be interpreted in one way.

There is also a risk of misunderstanding the questions or a lack of respondent involvement. In this work, this was eliminated by face-to-face interviewing.

Sometimes survey results may be false because patients want to present a better version of themselves.

Moreover, in survey research there is a risk of over-representation—respondents may include too many people with specific characteristics in relation to the surveyed population.

### 2.4. Statistical Analysis

The relationship between diet and oral cancer risk was analyzed using a logistic regression model. The odds ratio (OR) was used to estimate the risk of developing oral cancer.

The regression model used the following:

- A dependent variable as a grouping variable (membership in the study group (1) and control group (0));
- An independent variable, referred to as a predictor (the individual answers in the FFQ survey covering the frequency of consumption of specific products).

If OR ≈ 1, the chance of the disease occurrence in the study and control groups is similar. An OR < 1 indicates that the chance of developing the disease is lower in the study group (compared to the control group). An OR > 1 means that there is a greater chance of developing the disease in the study group (compared to the control group). A confidence interval was assumed for the obtained results with an assumption of 95% probability (95% CI). This means that each determined OR value has a 95% probability of falling within the specified confidence interval. The results with a significance level of *p* ≤ 0.05 were considered statistically significant. The analyzes were performed using the JASP statistical package.

## 3. Results

In total, 165 people took part in this study. The study group included 85 people with oral cancer (42% women and 58% men). The control group consisted of 80 healthy people (52% women and 48% men). The people in the control group had a higher level of education than the people with oral cancer. In the study group, more people performed physical work—42%; in the control group, it was 34%. The rest were white-collar workers. The data about the study participants are presented in Table 2.

The average age among the women in the study group was 59.9 years and 61.2 years among the men. In the study group, 28% of patients declared that exposure to carcinogenic factors was present. Among healthy people, this percentage was 25%. The addiction to smoking cigarettes and tobacco products affected 68% of the study group in the past. At the time of completing the survey, 12.5% of patients confirmed that they still smoked cigarettes. In the control group, this percentage was 35.5% and 18%, respectively. The most common cancer among the research group was tongue cancer, followed by cancer of the floor of the mouth and cheek. The average frequency of meat consumption in the study group was 3.6, which means that respondents ate meat more than a few times a month. In the control group, this value was 2.9, which is less than a few times a month.

In the study group, the non-response rate was 3%; in the control group, all respondents responded.

High meat consumption as a risk factor for the development of oral cancer was analyzed in three variants:

A. High total meat consumption as a risk factor;

B. High consumption of red meat as a risk factor;

C. High consumption of thermally processed meat products as a risk factor.

### 3.1. High Total Meat Consumption as a Risk Factor for Oral Cancer

The data presented in Table 3 show that frequent consumption of meat products and offal, as well as various types of sausages and bacon, is a significant risk factor for oral cancer (meat products: OR = 2.23; 95% CI = 1.42–3.50 and sausages: OR = 1.76; 95% CI = 1.06–2.94). The consumption of other types of meat turned out to be statistically insignificant. Bold results are statistically significant.

### 3.2. High Consumption of Red Meat as a Risk Factor for Oral Cancer

In the next analysis, the consumption of red meat, sausages and bacon was used as a variable. The calculations show that consumption of red meat is a statistically significant (*p*^1^ < 0.001) risk factor for developing oral cancer (OR = 2.47; 95% CI = 1.59–3.81). Taking into account both red meat and sausages and bacon, the consumption of the second group of products (i.e., sausages and bacon) turned out to be a statistically significant and stronger risk factor (OR = 2.06; 95% CI = 1.36–3.13). However, it should be noted that the value of the lower confidence interval (CI down^2^) is close to 1, i.e., the chance of disease occurrence in the study and control groups is similar, which indicates the need to conduct further research on a larger group of people to confirm or negate this result. Bold results are statistically significant.

The analysis of the data in Table 3 and Table 4 shows that if red meat dominates in the diet, it may be a risk factor for oral cancer. However, when using a mixed diet, rich in both red meat and other meat products, the consumption of processed products (meats, sausages and bacon) becomes a stronger risk factor for disease.

Smoking tobacco products is by far the most important risk factor for oral cancer. Table 5 presents the results of the analysis of the risk of this cancer in people following a diet rich in red meat and other processed meat products (sausages and bacon), taking into account smoking cigarettes and other tobacco products as an additional carcinogenic factor. Based on the data obtained, it can be concluded that, in people who smoke cigarettes, the consumption of red meat and other meat products (sausages and bacon) are statistically significant (*p* < 0.05) risk factors for developing oral cancer. For both products, the estimated risk was at a similar level as it was for a diet rich in red meat (OR = 1.69; 95% CI = 1.00–2.86) and for the consumption of sausages and bacon (OR = 1.81; 95% CI = 1.17–2.81). A comparison of the data contained in Table 3 and Table 5 shows that in people who smoke cigarettes, the estimated risk increased from 1.42 to 1.69 for the factor of consuming red meat, which may indicate a synergistic effect of cigarette smoking and a diet rich in meat on the development of oral cancer. Bold results are statistically significant.

Table 6 showed that sausage consumption remains a significant risk factor (*p* = 0.002) for oral cancer, if the consumption of red meat and sausages and the additional factor of drinking alcohol are taken into account. The odds ratio of getting sick (OR = 2.12) when consuming this product is slightly higher than in the case of the model that only took into account the consumption of sausages (OR = 2.06) without using alcohol as an additional factor (Table 5). Alcohol is a solvent for many compounds, including PAHs and HAAs, which may result in their better absorption in the gastrointestinal tract and an increase in exposure to the harmful effects of these carcinogenic compounds.

### 3.3. High Consumption of Thermally Processed Meat Products as a Risk Factor for Oral Cancer

Another aim of the statistical analysis was to estimate the risk of developing oral cancer depending on the consumption of meat thermally processed in various ways. The results are presented in Table 7. Bold results are statistically significant.

In the statistical analysis above, certain parameters—consumption of smoked, fried, roasted, boiled, and grilled meat—were correlated with each other as dependent factors occurring together in the diet. The statistical analysis, which assumed that the diet consisted of thermally processed meat in various ways, showed that only the consumption of smoked meat was a statistically significant factor (*p* = 0.010) in the risk of developing oral cancer (OR = 1.69; 95% CI = 1.13–2.51). However, in statistical models that considered each parameter separately, smoked, fried, baked, and boiled meat showed a positive correlation with the occurrence of oral cancer. Only grilled meat did not show a statistical significance.

When smoking cigarettes is an additional factor, the consumption of smoked meat products is a statistically significant (*p* = 0.011) carcinogenic factor, and smoking tobacco only slightly increases the estimated odds ratio (OR) (from 1.69 in non-smokers to 1.77 in smokers).

### 3.4. The Impact of Fruit and Vegetable Consumption on the Risk of Oral Cancer

Other dietary components included in the statistical analysis of factors leading to the development of or protecting against oral cancer are fruits and vegetables. The results of the analysis, which are presented in Table 8, confirmed that vegetable consumption is a statistically significant (*p* = 0.018) protective factor against cancer (OR = 0.62; 95% CI = 0.42–0.92). Contrary to expectations, fruit consumption turned out to be a significant (*p* = 0.004) risk factor for oral cancer (OR = 1.85; 95% CI = 1.22–2.80). The consumption of other products, i.e., dried fruit and juices, can be classified as protective factors (OR < 1), although this relationship does not show a statistical significance. Bold results are statistically significant.

The analysis, which only took into account the role of fruit consumption in the development of oral cancer, confirmed that fruit consumption is a significant (*p* = 0.036) risk factor for the disease (OR = 1.46; 95% CI = 1.02–2.07). The consumption of dried fruit turned out to be a significant (*p* = 0.037) protective factor (OR = 0.72; 95% CI = 0.53–0.98). The obtained results indicate no effect of fruit juice consumption on the development of oral cancer.

## 4. Discussion

### 4.1. Consumption of Meat and Meat Products as Risk Factors in the Etiopathology of Oral Cancer

Tobacco smoking, alcohol abuse, and papilloma virus infection are research-confirmed factors that increase the risk of cancer in the head and neck [8]. Although cancer is classified as a diet-related disease, the influence of diet on the development of oral cancer has so far been poorly understood [5,32,33]. The frequent consumption of certain products, including red meat and its products, is a risk factor for the development of gastrointestinal cancer [34,35,36].

A statistical analysis of the obtained data showed that frequent consumption of processed meat (cured meats, liver, blood sausage, brawn, pâtés, sausages and bacon), as well as red meat in general (pork, beef, and veal) is an important risk factor for developing cancers of the first section of the digestive tract—the oral cavity. Other authors have obtained similar results [32,37,38], but there are also studies that do not confirm this relationship [39,40,41]. The research conducted by Gupta et al. showed that the frequent consumption of red meat (at least once a week) is a factor of increased risk of OCCs [32]. Li et al. confirmed that a diet rich in red or processed meat may increase the risk of nasopharyngeal cancer [37], and a multicenter study conducted in China showed that the increased consumption of total meat and processed meat increases the risk of head and neck cancer [38]. According to the results of other case-control studies, the consumption of processed meat may be a carcinogenic factor, while the consumption of red meat does not influence the risk of developing oral cancer [39,40]. On the other hand, a cohort study conducted in the Netherlands showed that there is a positive correlation between the consumption of processed meat and the development of head and neck cancers, including cancer of the oral cavity but not of the throat and larynx, although there was no effect of the consumption of red meat to an increase in disease incidence [39]. The meta-analysis of research results conducted by Xu et al. also proves that consuming large amounts of processed meat significantly increases (by 91%) the risk of developing oropharyngeal cancer but no such relationship was observed when the statistical analysis included the consumption of red meat, white, or total meat [33].

The differences in research results regarding the impact of eating red meat on the risk of developing cancer, including oral cancer, may be due to the fact that the studies were conducted in different countries in different regions of the world. The dietary preferences of people living in these countries and the consumption of red meat from different animal species and even from different muscles, as well as the different methods of the thermal processing of meat, may affect the heme iron content [41]. Heme iron may contribute to the formation of carcinogenic N-nitroso compounds from nitrates (III) produced endogenously. Moreover, it can cause DNA damage by catalyzing oxidative stress [3,41].

A review of the literature on the subject, including 72 meta-analyses and 20 original publications, regarding the impact of a diet rich in meat on the development of cancer showed that the consumption of red meat is a risk factor for overall cancer mortality (including non-Hodgkin’s lymphoma, bladder, breast, intestinal, esophagus, stomach, lung, and nasopharynx cancer), although it does not clearly confirm that red meat influences the development of oral cancer. However, the result of this review strongly indicates that the consumption of processed meat is a factor that significantly increases the risk of overall cancer mortality, including oropharyngeal cancer [42].

Just like our own research showed, the research by Yan et al. showed a statistically significant relationship between meat consumption (at least three times a week) and tongue cancer, but only in cigarette smokers. Moreover, a synergistic relationship between cigarette smoking and meat consumption has been observed [40]. The results obtained in this study (Table 3 and Table 5) do not indicate such a strong role for smoking as in the study by Yan et al. [40], although it was noted that, in people who smoked cigarettes, the estimated risk of developing cancer increased from 1.42 to 1.69 for the factor of eating red meat. People who smoke cigarettes are exposed to many carcinogenic compounds from tobacco smoke, including PAHs and nitrosamines. The consumption of meat and meat products, i.e., products with a high fat content, probably increases the accumulation of these compounds in the human body, intensifying the processes of carcinogenesis [6,43,44].

The impact of a diet rich in meat, especially red meat, on the incidence of oral cancer is still not fully understood. In addition to the opinions on the harmfulness of consuming processed meat presented in the discussion, there are also studies in the literature whose results indicate that consuming meat products may protect against the development of benign head and neck cancers [45,46].

### 4.2. Consumption of Thermally Processed Meat and the Risk of Oral Cancer

According to the International Agency for Research on Cancer, thermally processed meat is a direct human carcinogen [3]. Heat processing improves the digestibility and flavor of the meat, but can also lead to the formation of carcinogens, including HAAs and PAHs. Most of these compounds are formed during high-temperature meat processing, such as grilling, frying, and roasting meat, as well as during meat preservation by smoking [47].

Based on a comprehensive systematic review and a meta-analysis of long-term studies investigating the relationship between a diet rich in meat and the development of various types of cancer, Farvid et al. showed that there is a statistically significant relationship between the consumption of thermally processed meat and an increase in the risk of cancers of the breast (by 6%), large intestine (18%), colon (21%), rectum (22%), and lung (12%) [48]. The above-mentioned cancers are mostly of epithelial origin, like OCCs, and therefore may have a similar etiology.

The term “thermally processed meat” is very broad. Meat products often consumed by inhabitants of particular continents may differ due to different culinary traditions and preferences for specific methods of preparing dishes with meat from different animals. A review of the literature shows that a diet rich in meat products subjected to specific heat treatment may influence the type of cancer lesions developing in the consumers. Eating fried foods is a risk factor for stomach, rectal, and colon cancer. Research has shown a moderately increased risk of oropharyngeal cancer in men with a diet rich in fried foods, as well as a risk of laryngeal cancer, especially in overweight people [5].

Maso et al. proved that there is a statistical relationship between the consumption of fried meat and an increased risk of cancer of the oral cavity, throat, and esophagus. The study shows that 20% of patients ate fried foods every day [49]. Research conducted in Uruguay shows that more frequent consumption of roasted or boiled meat doubles the risk of developing cancers of the upper digestive tract and respiratory system [50]. Moreover, it has been proven that there is a relationship between the cancer risk of these two areas and the total content of heterocyclic aromatic amines in food estimated on the basis of the FFQ [50].

The presented data from the literature show that the relationship between the consumption of meat products and the risk of developing oral cancer was usually considered in relation to the consumption of processed meat in general, possibly prepared in a certain way.

The research conducted as part of this work, one of the goals of which was to answer the question whether the consumption of thermally processed meat is one of the risk factors for oral cancer, took into account, unlike other published works, meat products prepared in five different ways, most often used in Polish homes. In Poland, it is common to eat meals prepared at home in the manner mentioned above. A statistical analysis of the responses of respondents completing the FFQ questionnaire showed that the frequent consumption of fried, roasted, smoked, and boiled meat increases the risk of OCCs, with the strongest risk factor being the consumption of smoked meat. No such relationship was found for the consumption of grilled meat.

The obtained results regarding the role of fried meat as a risk factor for oral cancer are consistent with the results described in the literature [5,32]. The processes of frying and roasting meat require the use of high temperatures. Increasing the temperature from 200 to 250 °C may result in a 6–7-fold increase in the concentration of mutagenic and carcinogenic compounds in meat. Therefore, eating fried products may be an important source of consumer exposure to compounds harmful to health [51].

A study conducted in Brazil showed that frequent consumption (at least four times a week) of grilled meat increases the risk of oral cancer. No such relationship was found in the group of people eating grilled meat once a month or less often [52]. The results conducted in this study did not confirm that eating grilled products increases the risk of oral cancer. In Poland, grilled dishes are eaten occasionally, mainly in the summer season. Therefore, the obtained result corresponds to the observation made by a group of researchers from Brazil, i.e., occasional consumption of grilled meat does not pose a risk of developing oral cancer.

No data have been found in scientific databases on the impact of a diet rich in smoked products on the development of oral cancer, although epidemiological studies indicate a correlation between the increased incidence of gastrointestinal cancer, especially stomach cancer, and frequent consumption of smoked food [53]. A particularly high increase in the incidence of stomach cancer was observed in the population of people consuming smoked meat products for their own use (at home). The statistical analysis of the survey data showed a strong relationship (*p* < 0.001) between the consumption of smoked meat and the risk of oral cancer (OR = 1.98; 95% CI = 1.41–2.79). Smoked products may contain large amounts of carcinogenic PAH compounds. The concentrations of BaP and PAH4 in smoked meat products available on Polish markets may reach 6 ng/g and 36 ng/g of the product, respectively. The greatest number of these compounds was determined in sausages and pork hams smoked according to traditional Polish recipes [54].

### 4.3. Fruit and Vegetable Consumption and the Risk of Oral Cancer

A statistical analysis of the frequency of the consumption of fruit and vegetables, as well as fruit and vegetable juices and dried fruit, by study participants led to unexpected results. The multivariate regression, determined with the assumption that all the above-mentioned products (Table 8) are predictors of OCCs, showed that the consumption of vegetables has a significant protective role (*p* = 0.018), while the consumption of fresh fruit unexpectedly turned out to be a risk factor. The consumption of dried fruit and juices was statistically insignificant. 

In a statistical model in which vegetables were missed and only the consumption of fresh and dried fruit and fruit juices was taken into account, it was confirmed that the consumption of fresh fruit is a significant (*p* = 0.036) risk factor for the development of oral cancer. Dried fruit turned out to be a protective factor (*p* = 0.037), and the role of the juices remained neutral. 

A review of the literature shows that a diet rich in vegetables, e.g., a vegetarian diet, may reduce the risk of various cancers, including cancers of the colon, prostate, and breasts. Some vegetables, e.g., lettuce, broccoli, cabbage, onion, and garlic, can effectively protect against cancer of the mouth, throat, esophagus, and stomach [55]. The studies by Bradford-Bella et al. have shown that eating vegetables and green salad at least once a week reduces the concentration of an oral cancer marker (CD44) in saliva and improves the prognosis of oncological patients [56]. These reports are consistent with the results of this study, indicating the protective role of vegetable consumption in the development of oral cancer.

A meta-analysis of the results of case-control studies conducted in Mediterranean countries on the consumption of vegetables and fruits confirmed the protective role of vegetables, especially raw vegetables, in relation to the risk of developing some common epithelial cancers [57]. For the risk of developing cancer of the larynx, oral cavity, and pharynx, the estimated OR was 0.2, which was lower than that determined in this study (0.62; Table 8). The differences in the OR values may result from the different dietary habits of people surveyed in Mediterranean countries and Poland.

Popular vegetables in Poland include garlic and onion. They are a source of biologically active sulfur compounds, amino acids, vitamins, and trace elements. Research confirms that consuming onion and garlic is a protective factor against the development of various cancers, including cancer of the oral cavity and pharynx, as well as the larynx, esophagus, and large intestine [58].

Eating yellow vegetables such as carrots, pumpkin, sweet potato, and corn, which are high in β-carotene [30,58], as well as cruciferous vegetables, more than once a week has been proven to reduce the risk of oral cancer [30]. Cruciferous vegetables (cabbage, cauliflower, broccoli, brussels sprouts, and turnips) are the products commonly consumed in Poland at all times of the year. They are a rich source of organic sulfur compounds, glucosinolates, whose main degradation products (isothiocyanates and indoles) have anticancer properties. Perhaps the presence of a significant share of such vegetables in the respondents’ diet influenced the significant protective role of vegetables in oral cancer observed in our work. Isothiocyanates may generate stable thioureas through the reaction with the carcinogenic metabolites of HAAs, which are then removed from the body. In vivo studies confirm that the intake of cruciferous plants together with thermally treated meat may reduce DNA damage in human tissues and reduce the carcinogenesis caused by the compounds generated by meat frying [59]. Frequent consumption of these vegetables significantly reduces the risk of mouth and throat cancer, as well as other cancers (esophagus, colon, breast, and kidney) [36].

The data in the literature on the relationship between fruit consumption and the risk of oral cancer mostly indicate their beneficial impact on health. Eating just one portion of fruit or vegetables a day significantly reduces the risk of developing oral cancer [55,58]. Some studies indicate that frequent consumption of vegetables is a stronger factor (than consumption of fruit) in reducing the risk of developing cancer, especially of the upper gastrointestinal tract [57] including the oral cavity [60]. In a cohort study, a reduced risk of developing cancer was only reported for consuming whole, fresh fruit but not fruit juice [60]. These data are partially consistent with the results of the statistical analysis obtained in this study (Table 8), which also showed that the consumption of vegetables may protect against the development of oral cancer, while the consumption of fruit juices is not a statistically significant factor in this process. 

Based on an extensive review of the literature on the relationship between the risk of developing head and neck cancer and the intake of carotenoids, an important source of which are vegetables and fruits, a positive relationship between carotenoid intake and better survival rates in oncological patients was demonstrated [58]. Due to the availability of food products, vegetables rather than fruits were a more important source of carotenoids for the respondents surveyed in this study. Carotenoids have antioxidant and anti-mutagenic properties, as well as immune regulatory functions [57]. However, cohort studies conducted in the Netherlands did not confirm that α- and β-carotene, lycopene, lutein, and zeaxanthin from fruits and vegetables reduce the risk of OCCs [61].

Apples are very popular fruits in Poland. According to the data in the literature, the consumption of these fruits contributes to reducing the risk of developing various types of cancer, including oral cancer, but only when considered in a multifactorial model [62]. Another study did not find that total fruit consumption had a beneficial effect on the risk of colorectal cancer, although such an effect was noted for apple consumption alone [63]. Similarly, total fruit consumption did not reduce the risk of breast cancer but high citrus fruit consumption was a protective factor for the development of this cancer [64] and also reduced the risk of oropharyngeal cancer by 50% [65].

The statistical analysis carried out in this study showed that the consumption of dried fruit is a significant (*p* = 0.037) protective factor (OR = 0.72; 95% CI = 0.53–0.98) for oral cancer. This result is consistent with the data in the literature. Studies looking for a relationship between the consumption of dried fruit and the risk of developing squamous cell carcinoma have shown that an increase in the consumption of dried fruit is a significant (*p* = 0.0131) factor in reducing the risk of oropharyngeal cancer [66]. Consuming freeze-dried berries has been shown to have a protective effect on the development of oral cancer, as well as colorectal and prostate cancer [67].

The role of fruit and vegetable consumption in the development of oral cancer is not easy to interpret. Vegetables and fruits contain many antioxidant compounds, which paradoxically, in excess, can intensify the oxidation processes in the body. In addition to health-promoting compounds, these products also contain allergens, antivitamins, acids, and substances that block the absorption of nutrients, e.g., calcium and iron (phytates, oxalic acid, and fiber) [68]. Exceeding the daily requirement for certain compounds of plant origin may even increase the risk of certain cancers, especially in smokers [51].

Summing up the discussion on the impact of fruit and vegetable consumption on the risk of oral cancer, it is worth noting that the surveys that are often used to assess this relationship may lead to certain errors in estimating the effects of the impact. They may result from incorrect classification of the study and control groups, as well as from the fact that questionnaires always present the subjective assessment of the respondent. The diet of people with oral cancer may have changed compared to eating habits in earlier periods of life, and the frequent consumption of fruit by cancer patients declared in the questionnaire could have contributed to the increase in the risk of oral cancer noted in this study. The possible distortion of respondents is also worth mentioning, which is common in surveys on nutrition. This is due to the social expectation of a healthy lifestyle.

## 5. Conclusions

Overall, this study found a significant association between diet and oral cancer.

1. The high consumption of thermally processed meat, especially smoked, fried, roasted, and boiled, increases the risk of oral cancer. Such processes lead to the formation of carcinogenic and mutagenic polycyclic aromatic hydrocarbons and heterocyclic aromatic amines.
2. The high consumption of red meat, which includes pork and beef often consumed in Poland, is a risk factor for oral cancer.
3. The consumption of vegetables is a protective factor against oral cancer.

Based on the results, dietary guidelines can be developed to reduce the risk of oral cancer. Research on oral cancer is sparse. Most published studies focus on cancer of the upper aerodigestive tract, with no clear indication of oral cancer. Therefore, further research is needed to confirm the relationship between oral cancer and a diet rich in processed high-protein products with high concentrations of PAHs and HAAs.

## Figures and Tables

**Table 1:** Classification of food groups in the FFQ-6.

Food Group	Products
Fats	Oil Butter Margarine cubes (for baking or frying) and margarine in cups (for spreading) Sour or sweet cream Other animal fats, e.g., lard, bacon Mayonnaise and dressings, e.g., salad dressings
Fruits	Fruits, all types in general Dried fruit, e.g., raisins, apricots, figs, apples, and plums
Vegetables and grains	Vegetables, all types in general Potatoes, in various forms, e.g., boiled, roasted, French fries, potato pancakes, and potato dumplings Nuts, e.g., peanuts, hazelnuts, walnuts, almonds, pistachios, cashews, coconut, and chestnuts Seeds, e.g., pumpkin, sesame, sunflower, and wheat
Meat and fish	Cold cuts, e.g., ham, tenderloin, poultry, pork, beef, and mixed Sausages, various types, e.g., frankfurters and bacon Meat products and organ meats, e.g., liver, black pudding, brawn, and pâtés Red meat, e.g., pork, beef, and veal Poultry meat, e.g., chicken, duck, and turkey Game, e.g., wild boar, roe deer, and hare Lean fish, e.g., pollock, cod, perch, hake, and carp Oily fish, e.g., salmon, sardines, herring, and mackerel
Drinks	Fruit juices and fruit nectars Vegetable and fruit juices Sweetened drinks e.g., Fanta, Coca-cola, and Sprite Beer Wine and drinks Vodka and spirits
Method of preparing food	Fried meat Cooked meat Smoked meat Roasted meat Grilled meat
Vitamins and dietary supplements	-

**Table 2:** Descriptive characteristics of the control and study groups (mean values ± standard deviation).

	Study Group	Control Group
age [years]	67.5 ± 12.4	53 ± 20.1
weight [kg]	65.5 ± 16.6	80 ± 13
height [cm]	171 ± 9.9	170.5 ± 7.8
BMI [kg/m^2^]	22.3 ± 5.2	27.5 ± 5.3

**Table 3:** Results of logistic regression regarding the consumption of meat and meat products as predictors in the group of patients with oral cancer compared to the group of healthy people.

Statistical Variable	OR	95% CI down	95% CI upper	*p*
**Meat products and offal** e.g., liver, black pudding, brawn, and pâtés	**2.23**	**1.42**	**3.50**	**<0.001**
**Sausages—various types and bacon**	**1.76**	**1.06**	**2.94**	**0.030**
Red meat e.g., pork, beef, and veal	1.42	0.82	2.48	0.213
Poultry meat e.g., poultry meat, chicken, duck, and turkey	1.16	0.60	2.21	0.663
High-quality meat e.g., ham, tenderloin, poultry, pork, beef, and mixed	1.09	0.70	1.70	0.696
Game meat e.g., meat from wild boar, roe deer, quail, wild duck, and hare	0.50	0.23	1.07	0.075

OR—estimated odds ratio, CI—confidence interval, and *p*—significance level.

**Table 4:** Results of logistic regression regarding the consumption of red meat and meat products as predictors in the group of patients with oral cancer compared to the group of healthy people.

Statistical Variable	OR ^1^	95% CI down ^1^	95% CI upper ^1^	*p* ^1^	OR ^2^	95% CI down ^2^	95% CI upper ^2^	*p* ^2^
**Red meat** e.g., pork, beef, and veal	**2.47**	**1.59**	**3.81**	**<0.001**	1.60	0.97	2.65	0.065
**Sausages—various types and bacon**	**-**	**-**	**-**	**-**	**2.06**	**1.36**	**3.13**	**<0.001**

OR—estimated odds ratio, CI—confidence interval, ^1^—parameters concerning the statistical analysis taking into account the consumption of only red meat (without sausages), and ^2^—parameters concerning the analysis taking into account the consumption of red meat and sausages and bacon.

**Table 5:** Results of logistic regression regarding the consumption of meat and meat products as predictors, including cigarette smoking as an additional factor in the group of oral cancer patients compared to the group of healthy people.

Statistical Variable	OR	95% CI down	95% CI upper	*p*
**Sausages—various types and bacon**	**1.81**	**1.17**	**2.81**	**0.008**
**Red meat** e.g., pork, beef, and veal	**1.69**	**1.00**	**2.86**	**0.049**
**Smoking**	**4.54**	**1.81**	**11.39**	**0.001**

OR—estimated odds ratio, CI—confidence interval, and *p*—significance level.

**Table 6:** Results of logistic regression regarding the consumption of meat and meat products as predictors, including alcohol drinking as an additional factor in the group of patients with oral cancer, compared to the group of healthy people.

Statistical Variable	OR	95% CI down	95% CI upper	*p*
**Sausages**	**2.12**	**1.32**	**3.36**	**0.002**
Red meat	1.70	0.98	2.94	0.057
Beer	0.91	0.61	1.34	0.619
**Wine/drinks**	**0.48**	**0.28**	**0.82**	**0.007**
Vodka	0.99	0.97	0.58	0.965

OR—estimated odds ratio, CI—confidence interval, and *p*—significance level.

**Table 7:** Results of logistic regression regarding the consumption of various types of thermally processed meat as predictors in the group of patients with oral cancer.

Statistical Variable	OR	95% CI down	95% CI upper	*p*
**Smoked meat**	**1.69**	**1.13**	**2.51**	**0.010**
Fried meeat	1.49	0.99	2.23	0.055
Boiled meat	1.39	0.95	2.03	0.088
Roasted meat	1.24	0.80	1.92	0.335
Grilled meat	0.65	0.41	1.01	0.055

OR—estimated odds ratio, CI—confidence interval, and *p*—significance level.

**Table 8:** Results of logistic regression regarding fruit and vegetable consumption as predictors in the group of oral cancer patients compared to the group of healthy people.

Statistical Variable	OR	95% CI down	95% CI upper	*p*
**Fruit**	**1.85**	**1.22**	**2.80**	**0.004**
Dry fruit	0.78	0.56	1.08	0.131
**Vegetables**	**0.62**	**0.42**	**0.92**	**0.018**
Fruit juices	0.94	0.71	1.24	0.645
Vegetable juices	0.91	0.67	1.23	0.544

OR—estimated odds ratio, CI—confidence interval, and *p*—significance level.

## Data Availability

The data presented in this study are available on request from the corresponding author due to privacy.
